# The Book World of Medicine and Science

**Published:** 1899-01-28

**Authors:** 


### THE BOOK WORLD OF MEDICINE AND SCIENCE.

THE ERECTION OF A CONSUMPTIVE SANATORIUM FOR THE PEOPLE. By Dr. NAHM. Translated and published with an Introduction by WILLIAM CALDWELL, M.A., M.D. (Belfast: Mayne and Boyd. 1898. Price 2s. net.)

THIS little pamphlet will be read with interest and with profit by those who intend to busy themselves in the erection or planning of sanatoria for consumptives. Being written by the superintending physician of the sanatorium at Ruppertshain and translated by the physician to the Belfast Throne Consumption Hospital, the book naturally contains a great deal of practical information on the subject. Many questions are carefully considered, such as locality, size, class of patients to be admitted, arrangement of bedrooms, verandahs, &c., which require somewhat different treatment in such an institution as is here dealt with to that which obtains in ordinary hospitals. The book does not pretend to be more than a sketch, and even so far as it goes can hardly be taken to be an authoritative pronouncement on the various subjects on which it treats, but it is a very suggestive little pamphlet.

CATALOGUE OF PATENTED SANITARY APPLIANCES FOR HOSPITALS, PUBLIC INSTITUTIONS, AND PRIVATE DWELLINGS. (London: S. Maw, Son, and Thompson.)

THIS is a very smartly got up and well illustrated catalogue of sanitary fittings supplied by the publishers. All kinds of closets, latrines, urinals, baths, and lavatories are described and priced, as also are the various-patterned sinks which are used in hospital werk. The smoothly-glazed fireclay table for the post-mortem room, sloping towards the sink placed at its lower end, strikes us as being very good. The table is dished so that everything runs cleanly into the sink and one trap does for all. We think it would be still further improved if instead of the flushing tap it had an attachment for a short length of hose and jet provided with mixing taps, such as are seen in shampooing rooms, so that water could be delivered on the table at any temperature. The catalogue is likely to be very useful to those who intend to refit and repair hospitals and institutions.

CLEANLINESS IN CHILDREN. By ROSE PETTY, Associate of the Sanitary Institute. (London: Scientific Press. Price 2d.)

THIS is a little book written with the object of giving information to guardians, board school teachers, district visitors, and others whose work lies among the neglected and underfed children of the poor, in regard to the various forms and evil consequences of dirt. In fact, dirt is the text and cleanliness is the teaching. Much useful information is to be found in the book about both animal and vegetable parasites, and directions are given as to the best methods for getting rid of them. Directions are also given for the management of "diseases accompanied by scabbing," a rough and ready classification according to treatment which no doubt has its own proper utility for the class of readers to which the teaching is addressed, however opposed it may be to modern ideas. These diseases represent, it is said, "what is popularly recognised as 'eczema,'" and "their cause is mainly mechanical irritation of dirt or accumulated secretions," in reference to which it may be of interest to refer to the definition of eczema given in Malcolm Morris' recent work on skin diseases, a definition which "excludes all forms of inflammation of the skin caused by chemical or mechanical irritants."

Many useful directions are given as to the management of those minor troubles connected with the eyes and eyelids which, if neglected, are fertile causes of more serious disease. Under the head of personal cleanliness there are directions as to better clothing, care of the teeth, and other matters, and then come hints as to furniture, ventilation, drainage, &c., a list of disinfectants in common use, and finally a table of infectious diseases, mentioning their period of incubation, their symptoms, their consequences, the duration of their infection, and other points of interest. Altogether a very useful little manual for those who have to deal with children.

THE LOCAL GOVERNMENT ANNUAL FOR 1899. Edited by S. EDGECUMBE-ROGERS. (London: The *Local Government Journal* Office. Price 2s. 6d.)

THE 1899 edition of this annual again shows considerable additions. The directory portion gives the names and addresses of the chief officials of all corporations, county councils, boards of guardians, urban and rural district councils, county and borough asylums, &c., throughout the kingdom, as well as the vestries, district boards, public libraries, public parks, and city companies of London. The abstract of Local Government legislation of 1898 will be found of considerable service to officials, while a feature of last year's edition has been amplified and classified with the title of "An Encyclopædia of Local Government," for the use of members and officials of various public bodies. The additions this year include a succinct history of the Corporation of the City of London, and a recital of her rights and privileges; the law of drains and sewers put in brief and comprehensive form; figures of interest to the educational world; the latest results of purchasing gas undertakings, and a list of the gas companies bought since 1876, with details as to receipts and expenditure; how to deal with infectious diseases in rural districts; items about the Local Government Board; statistics dealing with local government finance; information respecting the London water supply and the companies' capital expenditure; notes about Parish Councils; points about the public health; facts about tramways traffic; a most comprehensive collection of vaccination and small-pox statistics; a record of the efforts made in the municipalisation of the water supply; three of the principal examples of municipalisation, and other items, while the information under the heads of last year has been in every case amplified and brought up to date. In the "Chief Legal Decisions of the Year 1897-8," officials will find a ready means of reference and a useful help.

HUMANE SCIENCE LECTURES. By Various Authors. (London: George Bell and Sons.)

THIS is a collection of a few lectures which have been delivered under the auspices of the Humanitarian League and the Leigh Browne Trust. Included in the list is one by Dr. Milne Bramwell on "Suggestion: its Place in Medicine and Scientific Research," and one by Peter Kropotkin on "Natural Selection and Mutual Aid," the former of which is a readable article, and stands out favourably amid its surroundings.

